# Effects of replacing fishmeal with cottonseed protein concentrate on growth performance, blood metabolites, and the intestinal health of juvenile rainbow trout (*Oncorhynchus mykiss*)

**DOI:** 10.3389/fimmu.2022.1079677

**Published:** 2022-12-21

**Authors:** Yang Liu, Shuwei Ma, Weihua Lv, Honghe Shi, Guangwen Qiu, Hongmiao Chang, Shaoxia Lu, Di Wang, Changan Wang, Shicheng Han, Hongbai Liu

**Affiliations:** ^1^ Heilongjiang River Fisheries Research Institute, Chinese Academy of Fishery Sciences, Harbin, China; ^2^ Key Laboratory of Aquatic Animal Diseases and Immune Technology of Heilongjiang Province, Harbin, China; ^3^ Wuxi Fisheries College, Nanjing Agricultural University, Wuxi, China; ^4^ Animal Science and Technology College of Northeast Agricultural University, Harbin, China; ^5^ College of Fisheries and Life Science, Dalian Ocean University, Dalian, China

**Keywords:** cottonseed protein concentrate, rainbow trout, growth, blood metabolites, intestinal health

## Abstract

Cottonseed protein concentrate (CPC) is a potential non-food protein source for fishmeal replacement in fish feed. However, a high inclusion level of CPC in diets may have adverse effects on the metabolism and health of carnivorous fish. This study aimed to investigate CPC as a fishmeal alternative in the diet of rainbow trout *Oncorhynchus mykiss* based on growth performance, blood metabolites, and intestinal health. Five isonitrogenous (46% crude protein) and isolipidic (16% crude lipid) diets were formulated: a control diet (30% fishmeal) and four experimental diets with substitution of fishmeal by CPC at 25%, 50%, 75%, and 100%. A total of 600 fish (mean body weight 11.24g) were hand-fed the five formulated diets to apparent satiation for eight weeks. The results showed no adverse effects on growth performance when 75% dietary fishmeal was replaced by CPC. However, reduced growth and feed intake were observed in rainbow trout fed a fishmeal-free diet based on CPC (CPC100%). Changes in serum metabolites were also observed in CPC100% compared with the control group, including an increase in alanine aminotransferase (ALT), a decrease in alkaline phosphatase (ALP), alterations in free amino acids, and reductions in cholesterol metabolism. In addition, the CPC-based diet resulted in reduced intestinal trypsin, decreased villus height and width in the distal intestine, upregulated mRNA expression levels of inflammatory cytokines in the intestine, and impaired gut microbiota with reduced bacterial diversity and decreased abundance of *Bacillaceae* compared with the control group. The findings suggest that the optimum substitution rate of dietary fishmeal by CPC for rainbow trout should be less than 75%.

## 1 Introduction

Fishmeal replacement is one of the dominant research fields in aquaculture due to the urgent need for reducing fishmeal to the maximum extent in aquafeed for sustainable aquaculture (1, 2). A variety of protein sources have been investigated as fishmeal alternatives, including terrestrial animal proteins, plant products, and single-cell protein sources (3–5). Among these alternative protein sources, plant proteins possess certain positive characteristics, including wide availability, high production, competitive price, relatively high protein content, and reasonably balanced amino acid profiles (6). Some plant feedstuffs have been widely used in aquafeed, including soy products, corn, wheat, and rapeseed (7, 8). However, most of the plant feedstuffs have nutritional limitations (e.g., adverse antinutrient and toxic effects), resulting in low substitution rates of fishmeal in the diets of carnivorous fish species (6, 9). More importantly, nearly all current plant production can be consumed by humans, resulting in food-feed competition (10). Competition for the use of plant-based ingredients in aquafeed also comes from livestock, agriculture sectors, and biofuel production (3, 10).

Cottonseed protein concentrate (CPC) is a novel non-food protein derived from cottonseed. It contains a high protein content (crude protein: 60-70%) with a relatively balanced amino acid profile and low levels of anti-nutrients (e. g., gossypol) (11). Previous studies have shown that CPC can partially replace fishmeal in the diets of carnivorous fish, but the optimum substitution rate of fishmeal varied among species. It was reported that substituting 12-36% fishmeal protein exhibited no negative effects on growth performance of hybrid grouper (♀*Epinephelus fuscoguttatus* × ♂*Epinephelus lanceolatu*) (12). A recent study on largemouth bass (*Micropterus salmoides*) revealed that the optimum substitution rate of dietary fishmeal should be less than 75% (13). However, excessive inclusion of CPC in diets may result in growth suppression, liver inflammation, and impaired intestinal histology (12–14).

Rainbow trout (*Oncorhynchus mykiss*) is a typical widely cultured carnivorous fish species, with an annual worldwide production of over 0.8 million tons (15). The dietary protein requirement for rainbow trout is generally over 40%, and high-quality fishmeal is still one of the primary protein sources in commercial trout feed (9). Soy product is currently the most dominant plant protein used in the diets of rainbow trout (10, 16). However, the nutritional limitations and competition from human food and livestock diets limit its further application in trout feed. CPC is a potential alternative protein source for rainbow trout. Previous studies have shown that there were no adverse effects on growth or intestinal histomorphology in rainbow trout when 10-50% dietary fishmeal was replaced by CPC (17). However, replacing dietary fishmeal in diets at higher inclusion levels by CPC for rainbow trout has not been reported. Therefore, the purpose of the present study was to investigate CPC as a fishmeal alternative at high replacement levels in the diets of rainbow trout based on growth performance, blood metabolites, and intestinal health.

## 2 Materials and methods

### 2.1 Diet

Five isonitrogenous (46% crude protein) and isolipidic (16% crude lipid) diets were formulated, including a control diet (C1, 30% fishmeal) and four experimental diets with graded levels of CPC, corresponding to the substitution rates of 25% (C2), 50% (C3), 75% (C4), and 100% (C5) for dietary fishmeal protein (**Tables 1**, **2**). The CPC was obtained from Xinjiang Jinlan Vegetable Protein Co. Ltd. (China). It contained 65% crude protein and 230mg/kg free gossypol. The feed was processed using a laboratory granulator (HKJ-218, Tongli Grain Machinery, China) and made into 1.5-mm (diameter) pellets. All diets were kept at 4°C for use after being dried in a ventilated oven (60°C, 1.5 h).

**Table 1:** Formulation and chemical composition of the experimental diets (% dry matter).

Diet	FM	CPC25%	CPC50%	CPC75%	CPC100%
Ingredients					
Fishmeal^1^	30.00	22.50	15.00	7.50	0.00
Black soldier fly meal	5.00	5.00	5.00	5.00	5.00
Cottonseed protein concentrate^2^	0.00	7.50	15.00	22.50	30.00
Corn protein concentrate	12.00	12.00	12.00	12.00	12.00
Soy protein concentrate	15.00	15.00	15.00	15.00	15.00
Wheat gluten meal	2.00	2.00	2.00	2.00	2.00
Wheat flour	19.58	19.38	19.23	19.03	18.83
Fish oil	12.00	12.00	12.00	12.00	12.00
Premix^3^	1.00	1.00	1.00	1.00	1.00
L-lysine^4^	1.00	1.20	1.35	1.55	1.75
DL-methionine^5^	0.30	0.30	0.30	0.30	0.30
Taurine^6^	0.50	0.50	0.50	0.50	0.50
Sodium alginate	1.00	1.00	1.00	1.00	1.00
Chloride choline^7^	0.60	0.60	0.60	0.60	0.60
BHT^8^	0.02	0.02	0.02	0.02	0.02
Approximate chemical composition					
Crude protein	46.08	46.00	45.89	45.82	45.74
Crude lipid	16.09	16.07	16.05	16.05	16.02
Gross energyMJ/kg	21.79	21.96	22.12	22.28	22.45

^1^Fishmeal: from TASA Fish Product Co., Ltd., Peru.

^2^Cottonseed protein concentrate: Xinjiang Jinlan Co. Ltd, China.

^3^Premix (mg/kg or IU/kg diet): VA 750000 IU, VD_3_ 200000 IU, VE 6000 mg, VK_3_ 2000 mg, VB_1_ 1200 mg, VB_2_ 1200 mg, VB_6_ 1200 mg, VB_12_ 8 mg, VC 21000 mg, D-calcium pantothenate 2000 mg, niacinamide 9000 mg, folic acid 370 mg, D-biotin 15 mg, nositol 10000 mg, MgSO_4_ 6000 mg, ZnSO_4_ 4000 mg, MnSO_4_ 2500 mg, CuSO_4_ 2500 mg, FeSO_4_ 2500 mg, CoSO_4_ 160 mg, Ca(IO_3_)_2_ 200 mg, Na_2_SeO_3_ 40 mg.

^4^L-lysine, 98%, Macklin Inc., Shanghai, China.

^5^DL-methionine, 99%, Macklin Inc., Shanghai, China.

^6^Taurine, 99%, Macklin Inc., Shanghai, China.

^7^Chloride choline, 50%, Jujia Biotech Co., Ltd, Shandong, China.

^8^BHT, butylated hydroxytoluene, >99.0%, Aladdin Biochemical Technology, Co., Ltd, Shanghai, China.

**Table 2:** Amino acid composition of the experimental diets (% dry matter).

Amino Acids	FM	CPC25%	CPC50%	CPC75%	CPC100%
Met	1.15	1.10	1.06	1.01	0.97
Cys	0.48	0.57	0.66	0.71	0.78
Lys	2.88	2.85	2.78	2.73	2.69
Thr	1.60	1.55	1.51	1.46	1.41
Ile	1.75	1.71	1.62	1.58	1.50
His	1.00	1.02	1.03	1.05	1.07
Val	2.01	1.98	1.96	1.93	1.90
Leu	3.52	3.48	3.45	3.41	3.37
Arg	2.45	2.74	3.08	3.34	3.70
Phe	1.91	1.97	2.08	2.14	2.22
Tyr	1.74	1.67	1.61	1.54	1.48
Glu	6.42	6.75	7.13	7.47	7.84
Asp	3.36	3.37	3.37	3.38	3.39
Gly	2.20	2.02	1.87	1.70	1.55
Ser	1.92	1.91	1.90	1.89	1.89
Ala	2.38	2.25	2.12	2.04	1.90
Pro	2.60	2.47	2.30	2.15	2.03

### 2.2 Fish and feeding trial

Rainbow trout used in the experiment were obtained from Agrimarine Industries Inc. (Benxi, China). After a three-week acclimation, a total of 600 healthy fish with similar size were selected and randomly distributed among 20 tanks (30 individuals/tank) with the five diets in quadruplicate. The feeding trial was conducted in a recirculating aquaculture system with a total water volume of 8 m^3^. During the feeding trial, juvenile rainbow trout were hand-fed to apparent satiation twice daily (8:30 and 16:00). Water temperature was controlled at 12.5°C~13.5°C, and the inflow rate was maintained at 0.4 m/s. Dissolved oxygen was kept above 8 mg/L with a water change of 15–20% daily and a photoperiod of 12L: 12D. The feeding trial lasted eight weeks.

### 2.3 Sample collection

At the end of the feeding trial, all fish were weighed in batches with measurement for body lengths after anaesthetization with MS-222 (200 mg/L). Five fish from each tank were randomly selected for blood sampling and further serum samples. The viscera and liver of each fish were also weighed for the calculation of viscerosomatic index and hepatosomatic index. Afterwards, gut contents, intestine, and liver samples were collected. All gut contents and liver samples were stored at liquid nitrogen until further analysis. Three intestine samples were also kept in liquid nitrogen, and another two intestine samples, including proximal intestine and distal intestine, were stored in 10% neutral formalin (RightTech, Changchun, China) for histological analysis.

### 2.4 Biochemical analysis and histological analysis

Proximate composition and content of amino acids in ingredients, diets, and fish samples were determined by standard methods of AOAC (2012) (18). The proximate composition included moisture, crude protein, crude lipid, and ash. Serum biochemical indicators were measured using a BECKMAN CX4 automatic analyzer with commercial kits (Beckman, CA, USA). Targeted metabolomic analyses were conducted to determine free amino acids and free sterols using UPLC-MS/MS according to previously reported methods (19–21). Digestive enzyme activity in the intestine was measured using commercial kits (Nanjing Jiancheng Bioengineering Institute, China), including those for trypsin, lipase, and amylase. Proximal and distal intestine for histological analysis was examined by light microscopy after dehydration with ethanol, embedding in paraplast, and staining with hematoxylin and eosin.

### 2.5 Analysis of gut microbiota

Total DNA of bacteria was extracted from gut contents using a MP FastDNA Spin Kit (MP Biomedicals, Irvine, CA, USA) (*n* = 7). The quality of the extracted DNA was evaluated by 1% agarose gel electrophoresis. The V3-V4 hypervariable regions of the bacterial 16S rRNA were amplified by universal primers 338F and 806R using previously reported conditions (22). After purification, all amplicons were sequenced using an Illumina MiSeq platform (Illumina, San Diego, CA, USA).

For bioinformatic analysis of gut bacteria, assignment of qualified reads was performed based on operational taxonomic units (OTUs) at a similarity of 97% using the QIIME pipeline. (version 1.8.0) (23). The α-diversity was evaluated based on Sobs, Shannon, ACE, Chao, and Coverage indices. The β-diversity analysis was conducted by PCA using the Vegan package (Community Ecology Package) in the R language. Unique and shared OTUs between groups were analyzed by Venn diagrams. Differences of intestinal bacteria between groups were identified at the phylum and genus levels.

### 2.6 Gene expression

Total RNA was isolated from intestine (*n* = 6) using TRIzol Reagent (Invitrogen, Carlsbad, CA, USA) and then was reverse transcribed to cDNA (Takara, Dalian, China). Quantitative real-time PCR was conducted in a Real-Time PCR System (Applied Biosystems 7500) in triplicate using Takara TB Green Premix Ex Taq II (Tli RNaseH Plus) following the manufacturer’s instructions. Efficiencies of the specific primers used in this study were evaluated according to previously reported methods (24) (**Table S1**). The primers of the target and reference genes are listed in (43–46) (**Table 3**). Gene expression levels were calculated according to the threshold cycle (2^-ΔΔCt^) method (24).

**Table 3:** Specific primers for Real-time PCR.

Target gene	Primer	Sequence (5’→3’)	Accession number
β-actin**^43^**	F	ACAGACTGTACCCATCCCAAAC	AJ438158
	R	AAAAAGCGCCAAAATAACAGAA	
C3**^44^**	F	GGCCAGTCCCTGGTGGTTA	L24433
	R	GGTGGACTGTGTGGATCCGTA	
C4**^44^**	F	TCTACAACCCTACACAGCAAGTGAG	AJ544262
	R	TGCCCGCAGCATTAAAAATAG	
IL1β**^45^**	F	ACCGAGTTCAAGGACAAGGA	AJ223954
	R	CATTCATCAGGACCCAGCAC	
IL8**^45^**	F	CACAGACAGAGAAGGAAGGAAAG	AJ279069
	R	TGCTCATCTTGGGGTTACAGA	
TNF-α**^43^**	F	GGGGACAAACTGTGGACTGA	AJ277604
	R	GAAGTTCTTGCCCTGCTCTG	
IL10**^45^**	F	CGACTTTAAATCTCCCATCGAC	AB118099
	R	GCATTGGACGATCTCTTTCTTC	
TGF-β**^45^**	F	AGATAAATCGGAGAGTTGCTGTG	AJ007836
	R	CCTGCTCCACCTTGTGTTGT	
OCLN**^45^**	F	CAGCCCAGTTCCTCCAGTAG	GQ476574
	R	GCTCATCCAGCTCTCTGTCC	
TRIC**^45^**	F	GTCACATCCCCAAACCAGTC	KC603902
	R	GTCCAGCTCGTCAAACTTCC	
ZO1**^45^**	F	AAGGAAGGTCTGGAGGAAGG	HQ656020
	R	CAGCTTGCCGTTGTAGAGG	
CLD1**^46^**	F	GAGGACCAGGAGAAGAAGG	BK008768
	R	AGCCCCAACCTACGAAC	

C3, complement component 3; C4, complement component 4; IL1β, interleukin 1 beta; IL8, interleukin 8; TNFα, tumor necrosis factor alpha; IL10, interleukin 10; TGFβ, transforming growth factor beta; OCLN, occludin; TRIC, tricellulin; ZO-1, zonula occludens-1; CLD1, claudin-1.

### 2.7 Statistical analysis

Differences among the five groups were analyzed based on one-way ANOVA with Duncan’s multiple range test using SPSS 19.0 software (SPSS Inc., Chicago, IL, USA). Comparisons between two groups were analyzed by independent samples t-test using the same software. Significant differences were determined by *P*< 0.05.

## 3 Results

### 3.1 Growth and feed utilization

At the end of the feeding trial, no significant differences in growth performance were observed among the control group (FM), CPC25%, CPC50%, and CPC75% (*P* > 0.05). However, complete fishmeal replacement by CPC (CPC100%) resulted in reduced feed intake and growth (*P* < 0.05). There was a significant decrease of condition factor (CF) in CPC75% and CPC100% compared with other groups (*P* < 0.05). Reductions of viscerosomatic index (VSI) and hepatosomatic index (HSI) were also found in experimental fish fed diets containing graded CPC (*P* < 0.05). Survival rates in all groups were higher than 97%, with no significant difference (*P* > 0.05) (**Table 4**).

**Table 4:** Growth, feed utilization and survival of rainbow trout fed diets containing graded CPC (mean ± S.E.).

Item	FM	CPC25%	CPC50%	CPC75%	CPC100%
Initial body weight (g)	11.26 ± 0.12	11.24 ± 0.10	11.27 ± 0.03	11.17 ± 0.14	11.22 ± 0.04
Final body weight (g)	46.15 ± 0.81^b^	44.50 ± 1.03^b^	44.76 ± 0.17^b^	44.34 ± 0.83^b^	40.36 ± 0.43^a^
Weight gain (%)	309.88 ± 4.39^b^	295.85 ± 8.02^b^	297.11 ± 2.78^b^	297.03 ± 3.42^b^	260.62 ± 3.12^a^
SGR (%/day)	2.52 ± 0.02^b^	2.46 ± 0.04^b^	2.46 ± 0.01^b^	2.46 ± 0.02^b^	2.29 ± 0.02^a^
FR (%/day)	2.27 ± 0.04^b^	2.22 ± 0.03^b^	2.22 ± 0.03^b^	2.20 ± 0.02^b^	2.06 ± 0.02^a^
FCR	1.04 ± 0.01	1.04 ± 0.02	1.04 ± 0.01	1.03 ± 0.01	1.03 ± 0.02
CF (g cm^-3^)	1.51 ± 0.01^b^	1.54 ± 0.02^b^	1.56 ± 0.02^b^	1.43 ± 0.02^a^	1.39 ± 0.01^a^
VSI (%)	17.34 ± 0.28^c^	16.40 ± 0.36^b^	15.81 ± 0.40^ab^	15.52 ± 0.24^ab^	14.95 ± 0.26^a^
HSI (%)	1.50 ± 0.04^c^	1.48 ± 0.04^c^	1.33 ± 0.04^b^	1.30 ± 0.03^ab^	1.22 ± 0.04^a^
Survival rate (%)	99.17 ± 0.83	99.17 ± 0.83	99.17 ± 0.83	99.17 ± 0.83	97.50 ± 0.83

IBW, Initial body weight; FBW, Final body weight;

Weight gain (WG, %)=100×[(FBW – IBW) × IBW^−1^].

Specific growth rate (SGR, %/day)=100×(ln FBW – ln IBW)/days;

Feeding rate (FR, %/day)=100×FI/[days×(FBW − IBW)/2];

Feed conversion ratio (FCR)=FI/WG;

Condition factor (CF, g cm^-3^) = 100×body weight/(body length)^3^, n=16;

Viscerosomatic index (VSI, %) = 100×viscera wet weight/body weight, n=16;

Hepatosomatic index (HSI, %) = 100 ×liver wet weight/body weight, n=16;

Survival rate = 100×(final number of fish)/(initial number of fish).

values with different superscripts in the same column are significantly different (P<0.05).

### 3.2 Chemical composition of whole fish

No significant difference in whole-body composition was found among groups, including moisture, crude protein, crude lipid, and ash (*P* > 0.05) (**Table 5**).

**Table 5:** Chemical composition of whole-body fish fed diets containing graded CPC (mean ± S.E, *n*=8).

Item	Moisture	Crude protein	Crude lipid	Ash
**FM**	68.06 ± 0.44	17.14 ± 0.20	10.55 ± 0.09	1.54 ± 0.03
**CPC25%**	67.71 ± 0.57	17.69 ± 0.28	10.58 ± 0.11	1.60 ± 0.03
**CPC50%**	68.15 ± 0.46	17.35 ± 0.33	10.28 ± 0.08	1.53 ± 0.04
**CPC75%**	67.91 ± 0.67	17.15 ± 0.13	10.41 ± 0.10	1.57 ± 0.03
**CPC100%**	67.31 ± 0.34	17.89 ± 0.20	10.61 ± 0.14	1.60 ± 0.04

Values with different superscripts in the same column are significantly different (P<0.05).

### 3.3 Blood metabolites

An increase in serum ALT and a reduction of serum ALP were observed in groups with graded CPC (*P* < 0.05). There was also a significant decrease in serum TC of CPC100% compared with that in FM (*P* < 0.05) (**Table 6**).

**Table 6:** Effects of fishmeal replacement by cottonseed protein concentrate on serum chemistry of rainbow trout (Mean ± S.E., *n*=6).

Item	FM	CPC25%	CPC50%	CPC75%	CPC100%
ALT(U/L)	201.48 ± 5.17^a^	262.18 ± 10.35^b^	255.50 ± 3.16^b^	275.88 ± 14.97^bc^	306.93 ± 19.74^c^
GLU(g/L)	22.45 ± 0.46	22.98 ± 0.33	23.35 ± 0.64	23.40 ± 0.12	22.90 ± 0.36
ALP(U/L)	428.83 ± 22.25^d^	315.03 ± 22.96^c^	323.73 ± 36.78^c^	240.25 ± 20.18^b^	142.18 ± 7.27^a^
TP(g/L)	40.05 ± 0.44	40.83 ± 0.39	41.63 ± 1.01	41.58 ± 0.17	40.68 ± 0.64
ALB(g/L)	17.72 ± 0.33	17.98 ± 0.28	18.28 ± 0.38	18.38 ± 0.08	17.78 ± 0.32
TC(mmol/L)	10.14 ± 0.26^b^	10.31 ± 0.29^b^	10.20 ± 0.70^b^	9.16 ± 0.30^ab^	8.45 ± 0.33^a^
TG(mmol/L)	7.18 ± 0.57	6.96 ± 0.58	6.23 ± 0.34	7.24 ± 0.20	6.38 ± 0.33
HDL-C(mmol/L)	3.72 ± 0.12	3.73 ± 0.22	3.87 ± 0.14	3.44 ± 0.11	3.44 ± 0.13
LDL-C(mmol/L)	7.22 ± 0.15	7.32 ± 0.40	7.52 ± 0.24	6.71 ± 0.23	6.67 ± 0.25
TBA(μmmol/L)	4.78 ± 0.53	5.58 ± 0.92	5.66 ± 0.33	5.54 ± 0.63	5.12 ± 0.43

TP, total protein; ALB, albumin; TC, total cholesterol; HDL-C, high density lipoprotein cholesterol; LDL-C, low density lipoprotein cholesterol; TG, triglyceride; TBA, total bile acid; GLU, glucose; ALT, alanine aminotransferase; ALP, alkaline phosphatase;

Values with different superscripts in the same column are significantly different (P<0.05).

Analysis of free amino acids in the serum showed that lysine, serine, proline, glutamic acid, methionine, tyrosine, and cysteine in CPC100% were significantly lower than those in FM (*P* < 0.05). However, valine, leucine, histidine, arginine, and tryptophan in CPC100% were significantly higher than those in FM (*P* < 0.05) (**Table 7**).

**Table 7:** Free amino acid in serum of rainbow trout fed different diets (Mean ± S.E., *n*=6).

Amino acid (μmol/L)	FM	CPC100%
Lysine	238.85 ± 4.03^b^	205.83 ± 9.93^a^
Glycine	1237.42 ± 77.53	1062.86 ± 51.43
Alanine	509.76 ± 21.49	501.62 ± 11.20
Serine	171.38 ± 11.55 ^b^	130.65 ± 3.83 ^a^
Proline	271.79 ± 32.64^b^	117.80 ± 10.66^a^
Valine	404.93 ± 8.64^a^	454.49 ± 15.78^b^
Threonine	195.80 ± 13.69	165.10 ± 17.15
Leucine	453.09 ± 16.09^a^	535.21 ± 25.02^b^
Isoleucine	95.74 ± 11.40	109.90 ± 10.99
Asparagine	51.87 ± 4.35	66.71 ± 8.44
Aspartic acid	48.79 ± 2.80	39.73 ± 2.91
Glutamine	322.60 ± 35.60	323.62 ± 19.57
Glutamic acid	148.36 ± 6.87^b^	112.81 ± 7.54^a^
Methionine	242.56 ± 23.44^b^	142.75 ± 3.63^a^
Histidine	182.13 ± 8.57^a^	312.95 ± 16.25^b^
Phenylalanine	164.39 ± 6.18	184.35 ± 6.60
Arginine	98.49 ± 3.18^a^	128.98 ± 6.96^b^
Tyrosine	107.48 ± 6.28^b^	84.31 ± 4.91^a^
Tryptophan	29.17 ± 1.77 ^a^	36.05 ± 1.84 ^b^
Cysteine	3.60 ± 0.12^b^	2.20 ± 0.08^a^

Values with different superscripts in the same column are significantly different (P<0.05).

Targeted metabolomics for free cholesterol in serum showed no significant difference in free cholesterol between FM and CPC100%. However, decreases in ergosterol, lathosterol, and cholestanol and increases in desmosterol and 7-dehydrocholesterol in CPC100% were observed compared with FM (**Table 8**).

**Table 8:** Free sterols in serum of rainbow trout fed different diets (Mean ± S.E., *n*=6).

Amino acid (nmol/L)	FM	CPC100%
Pregnenolone	6.76 ± 0.65	6.07 ± 0.29
Cholesterol 5α,6α-epoxide	270.47 ± 13.96	264.05 ± 3.33
Desmosterol	2995.46 ± 217.69^a^	3758.26 ± 61.17^b^
7-Dehydrocholesterol	6658.35 ± 551.79^a^	8578.67 ± 139.63^b^
Ergosterol	96.93 ± 8.92^b^	28.47 ± 1.61^a^
Lathosterol	1592.53 ± 235.85^b^	868.10 ± 74.58^a^
Brassicasterol	22.50 ± 5.87	19.83 ± 1.76
Cholesterol	2240990.41 ± 76364.64	2153811.15 ± 52256.36
Lanosterol	3531.64 ± 516.72	3248.41 ± 208.40
Campesterol	8147.26 ± 478.64	9506.76 ± 478.28
Cholestanol	7540.16 ± 556.70^b^	4615.27 ± 62.12^a^
β-Sitosterol	19876.29 ± 1672.66	24641.22 ± 2290.78

Values with different superscripts in the same column are significantly different (P<0.05).

### 3.4 Digestive enzyme activity in the intestine

**Figure 1:**
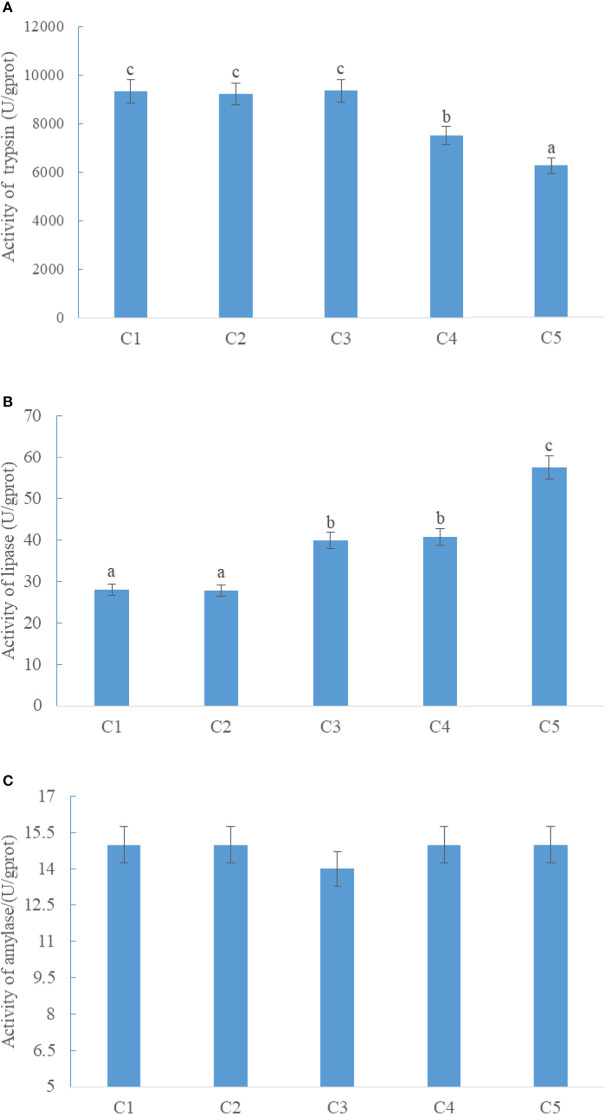
Effects of fishmeal replacement by cottonseed protein concentrate on the activity of intestinal trypsin **(A)**, lipase **(B)**, and amylase **(C)** in rainbow trout (Mean ± S.E.). Different letters on the bar chart indicate significant difference (*P <*0.05).

The activity of intestinal trypsin in CPC75% and CPC100% was significantly lower than that in FM (*p* < 0.05), and there were no significant differences among FM, CPC25%, and CPC50% (*P* > 0.05) (**Figure 1A**). An increase in activity of intestinal lipase was observed in groups with graded CPC, and the activity of intestinal lipase in CPC50%, CPC75%, and CPC100% was significantly higher than that in FM and CPC25% (*P* < 0.05) (**Figure 1B**). No significant difference in activity of intestinal amylase was observed among groups (*P* > 0.05) (**Figure 1C**).

### 3.5 Intestinal histology

**Figure 2:**
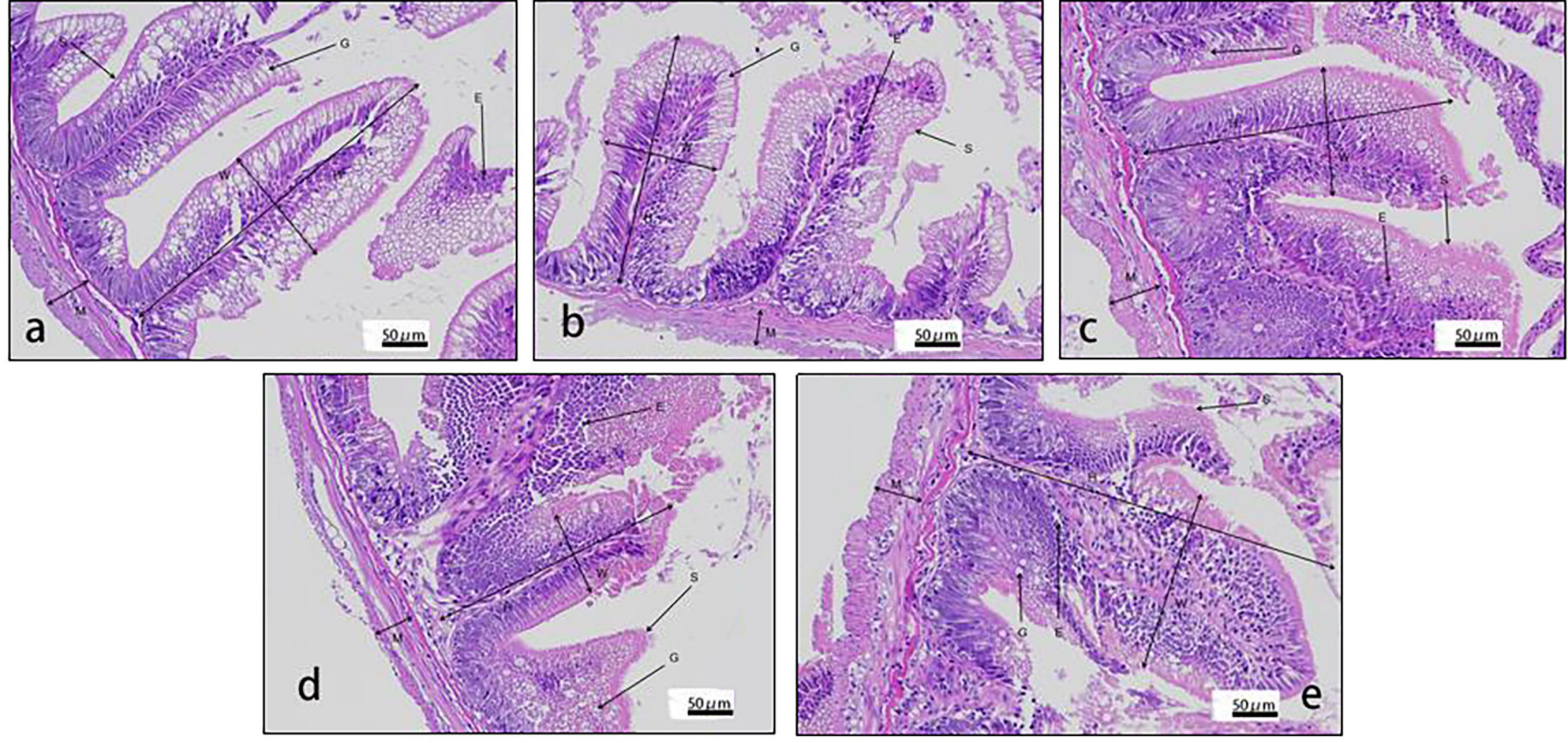
Histology of distal intestine in rainbow trout fed graded cottonseed protein concentrate. **(A)** FM; **(B)** CPC25%; **(C)** CPC50%; **(D)** CPC75%; **(E)** CPC100%. W-villus width, H-villus height, M-muscular layer thickness, S-striatum; G-goblet cells and E-epithelial cells.

**Figure 3:**
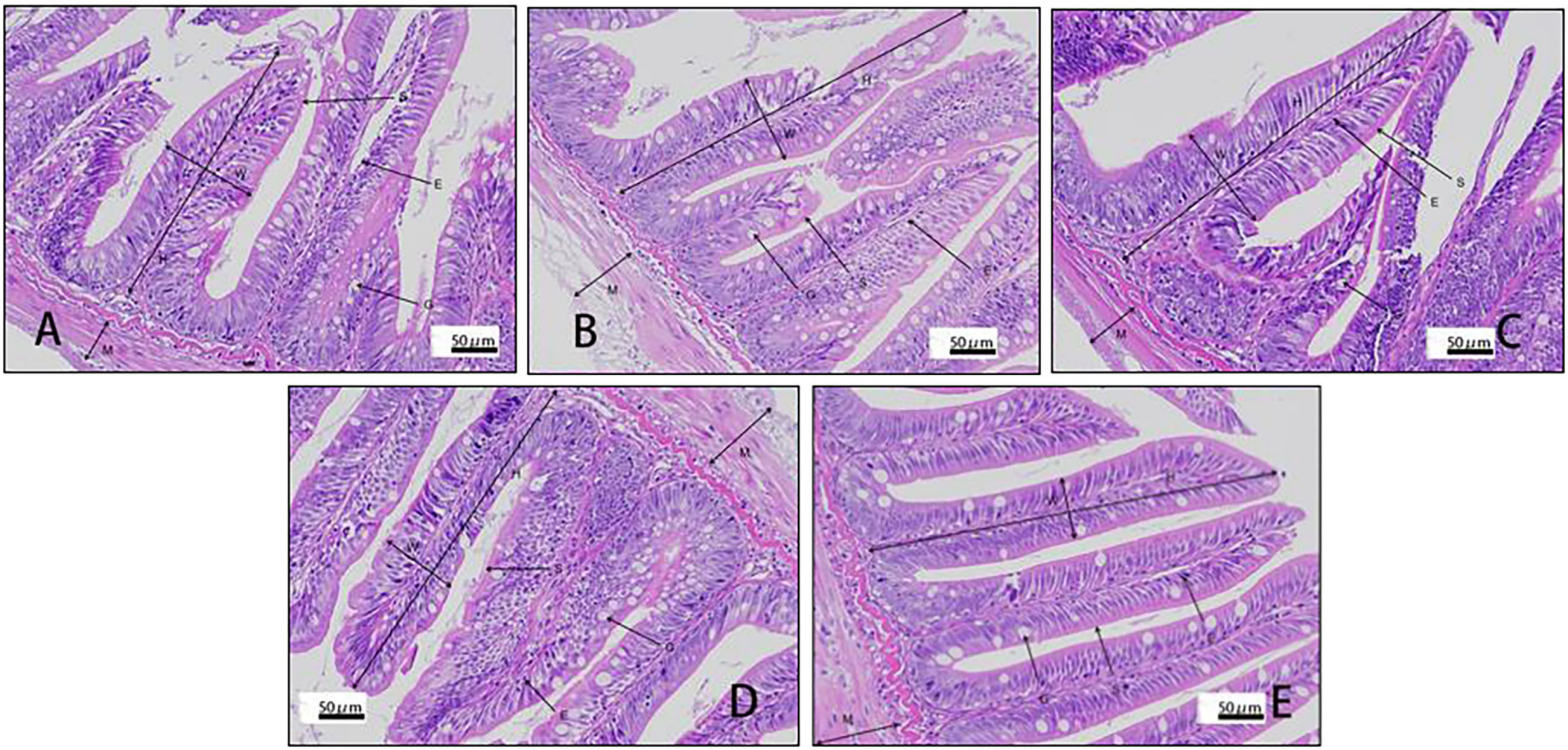
Histology of proximal intestine in rainbow trout fed graded cottonseed protein concentrate. **(A)** FM; **(B)** CPC25%; **(C)** CPC50%; **(D)** CPC75%; **(E)** CPC100%. W-villus width, H-villus height, M-muscular layer thickness, S-striatum; G-goblet cells and E-epithelial cells.

Villus height and width in the distal intestine decreased in groups with graded CPC, and a significant reduction was observed in CPC25%, CPC50%, CPC75% and CPC100% compared with FM (*P* < 0.05) (**Table 9**, **Figure 2**). However, no significant difference in muscular thickness of the distal intestine was observed between groups (*P* > 0.05) (**Table 9**, **Figure 2**). In addition, no significant differences in muscular thickness, villus height, or width in the proximal intestine were found among groups with graded CPC (*P* < 0.05) (**Table 10**, **Figure 3**).

**Table 9:** Effects of fishmeal replacement by cottonseed protein concentrate on histology of distal intestine in rainbow trout (Mean ± S.E.).

Item	FM	CPC25%	CPC50%	CPC75%	CPC100%
Villus height (μm)	924.90 ± 58.04^c^	834.43 ± 38.06^bc^	817.82 ± 16.07^bc^	743.02 ± 47.96^b^	558.86 ± 93.99^a^
Villus width (μm)	487.87 ± 39.40^b^	347.58 ± 16.86^a^	331.18 ± 13.79^a^	297.40 ± 24.01^a^	285.66 ± 11.14^a^
Muscular thickness (μm)	140.05 ± 6.33	170.54 ± 18.60	143.49 ± 13.16	144.65 ± 13.19	167.42 ± 11.12

Values with different superscripts in the same column are significantly different (P<0.05).

**Table 10:** Effects of fishmeal replacement by cottonseed protein concentrate on histology of proximal intestine in rainbow trout (Mean ± S.E.).

Item	FM	CPC25%	CPC50%	CPC75%	CPC100%
Villus height (μm)	980.71 ± 30.75	1084.60 ± 36.64	1002.18 ± 54.63	982.93 ± 31.51	1006.23 ± 56.60
Villus width (μm)	301.43 ± 9.30	290.80 ± 28.51	277.86 ± 17.57	316.33 ± 16.62	265.26 ± 7.18
Muscular thickness (μm)	168.20 ± 8.31	208.25 ± 18.54	172.88 ± 18.53	165.92 ± 4.74	183.71 ± 18.94

### 3.6 Expression of immune genes and tight junction protein genes in intestine

**Figure 4:**
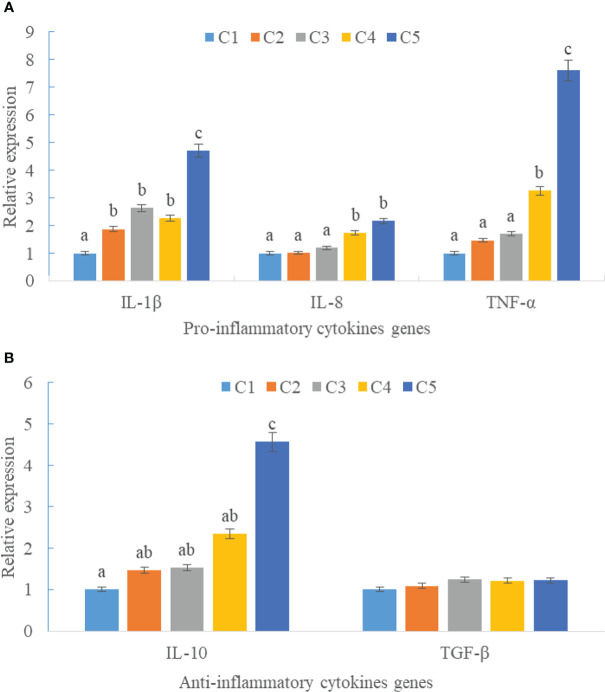
Effects of fishmeal replacement by cottonseed protein concentrate on the mRNA expression levels of inflammatory cytokines in intestine of rainbow trout (Mean ± S.E.). **(A)** Pro-inflammatory cytokines genes; **(B)** Anti-inflammatory cytokines genes. Different letters on the bar chart indicate significant difference (*P <*0.05).

**Figure 5:**
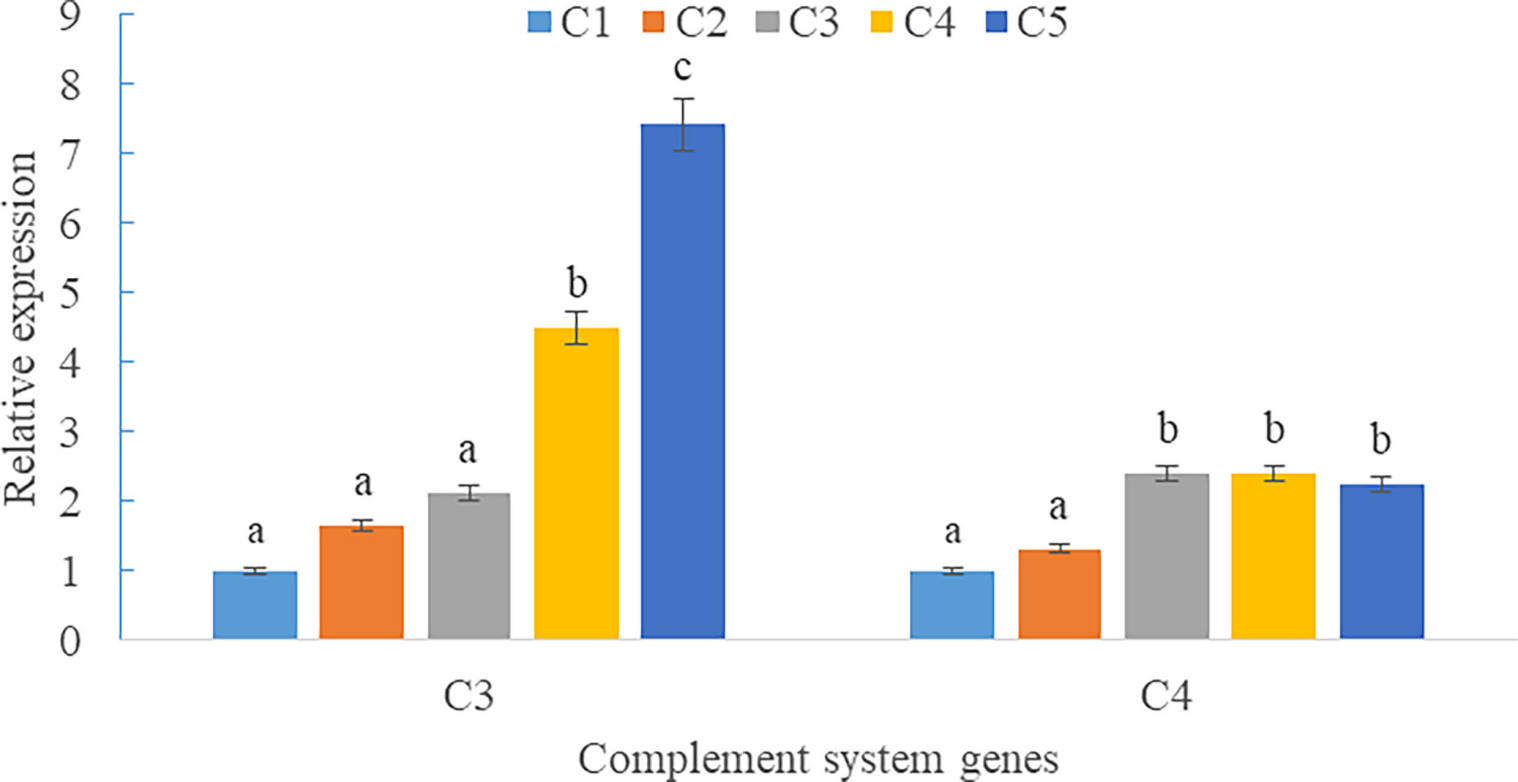
Effects of fishmeal replacement by cottonseed protein concentrate on the mRNA expression levels of complement system genes *c3* and *c4* in intestine of rainbow trout (Mean ± S.E.). Different letters on the bar chart indicate significant difference (*P <*0.05).

**Figure 6:**
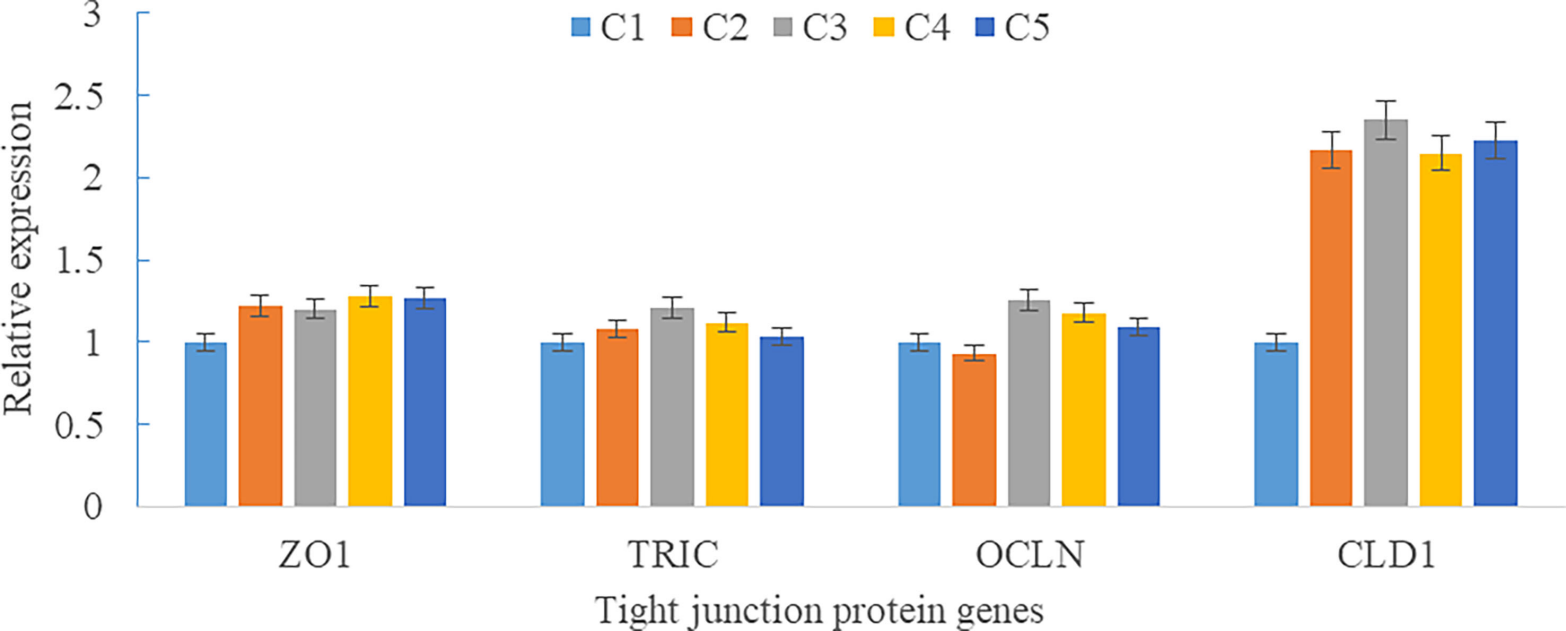
Effects of fishmeal replacement by cottonseed protein concentrate on the mRNA expression levels of tight junction protein genes in intestine of rainbow trout (Mean ± S.E.). Different letters on the bar chart indicate significant difference (*P <*0.05).

Upregulations in mRNA expression levels of inflammatory cytokines were observed in groups with graded CPC, including increases of IL-1β, IL-8, and TNF-α in CPC75% and CPC100% compared with FM, and an increase of IL-10 in CPC100% compared with other groups (*P* < 0.05) (**Figures 4A, B**). However, no significant difference in the mRNA expression level of TGF-β was found between groups (*P* > 0.05) (**Figure 4B**). Also, there was an increase in the mRNA expression level of complement system genes *c3* and *c4* in the intestine with graded CPC in diets (*P* < 0.05) (**Figure 5**). In addition, no significant differences in the mRNA expression levels of tight junction protein genes (ZO1, TRIC, OCLN, CLD1) in the intestine were observed between groups (*P* > 0.05) (**Figure 6**).

### 3.7 Gut microbiota

A total of 1961271 clean sequences were obtained from 35 samples (seven per group) with an average length of 416 bp. The rarefaction curves tended to saturation, indicating that the raw data can be used for further analysis (**Figure S2**). The analysis of alpha diversity indices showed that there were decreases in the Sobs, Shannon, Ace, and Chao indices with graded CPC in diets, including reductions of Sobs and Shannon indices in CPC50%, CPC75% and CPC100%, and decreases in the Ace and Chao indices in CPC25%, CPC50%, CPC75% and CPC100% compared with those in FM (*P* < 0.05) (**Table S2**). Sequencing coverage in each group was above 99%, and no significant difference was observed between groups (*P* > 0.05) (**Table S2**). In particular, there was a clear separation between FM and CPC100% based on the PCA plot (**Figure S2**).

**Figure 7:**
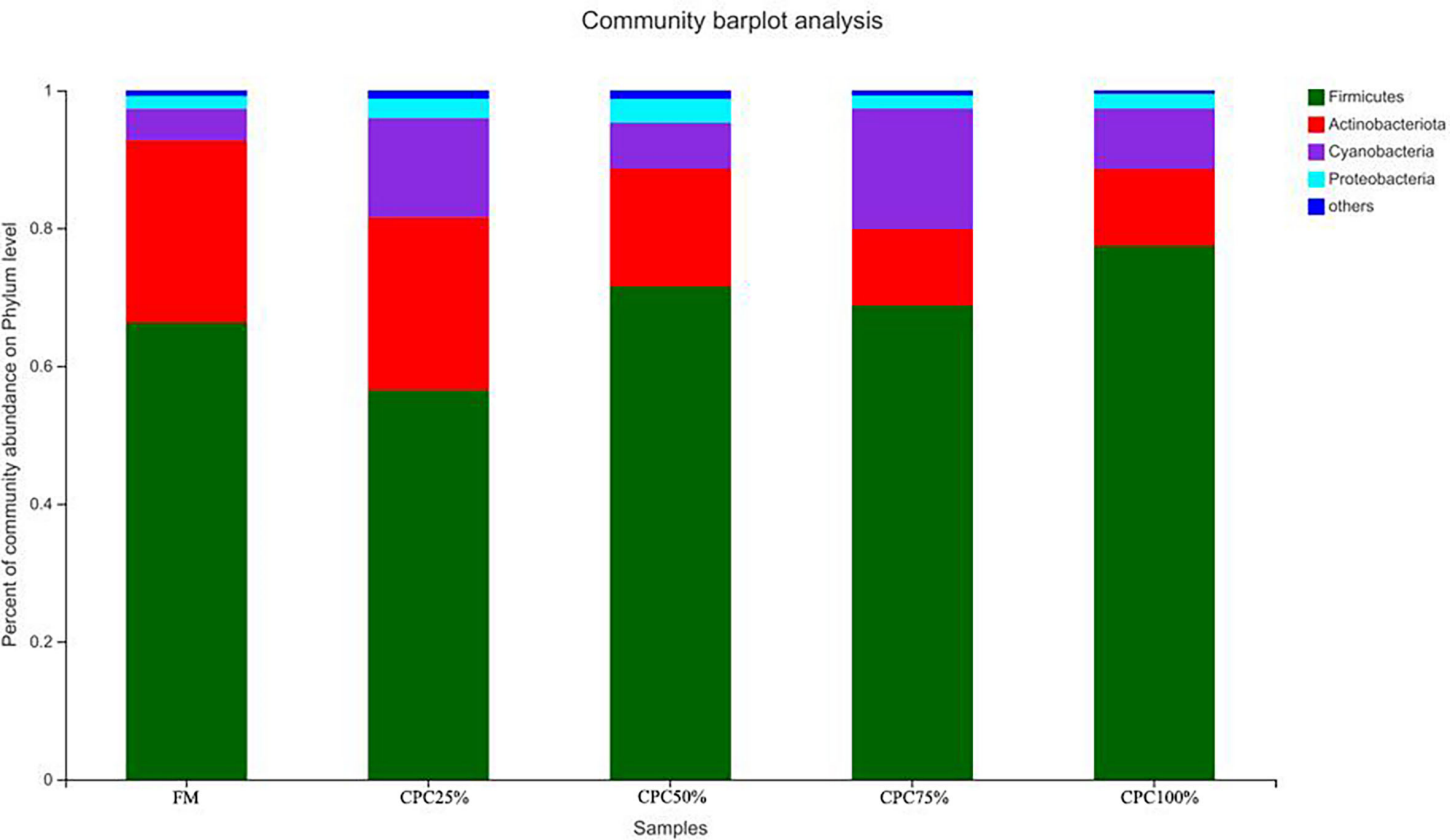
Effects of fishmeal replacement by cottonseed protein concentrate on relative abundance of intestinal microbes in juvenile rainbow trout at the phylum level.

**Figure 8:**
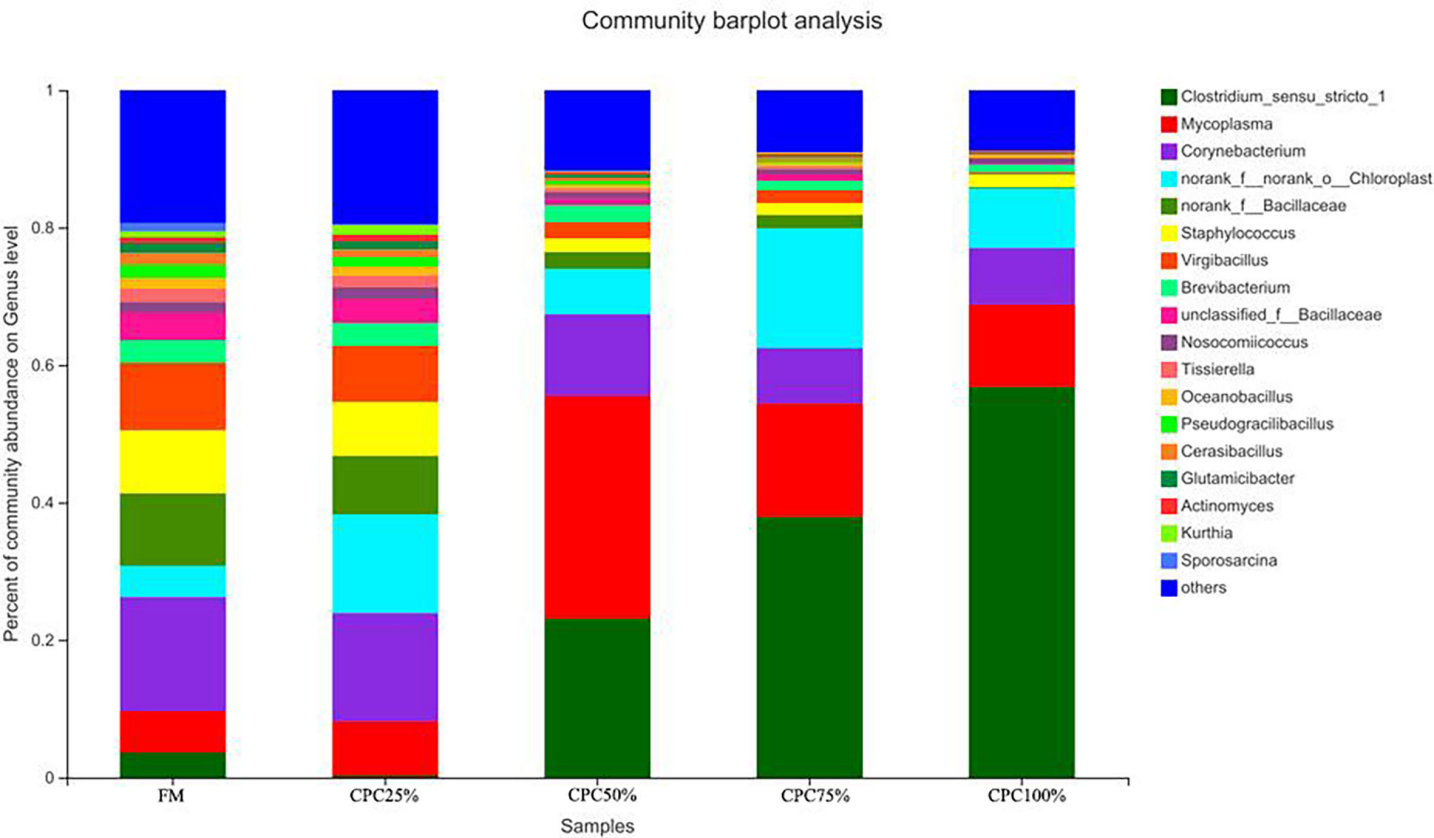
Effects of fishmeal replacement by cottonseed protein concentrate on relative abundance of intestinal microbes in juvenile rainbow trout at the genus level.

The Venn diagram analysis showed a total of 179 shared OTUs between groups, and the numbers of unique OTUs in FM, CPC25%, CPC50%, CPC75% and CPC100% were 61, 24, 44, 11, and 36, respectively (**Figure S3**). At the phylum level, fishmeal replacement by CPC in diets resulted in a decrease in Actinobacteriota and an increase in Firmicutes (**Figure 7**). At the genus level, there were decreases in *Corynebacterium*, *Staphylococcus, norank_f_Bacillaceae, virgibacillus, Brevibacterium*, and an increase in *Clostridium_sensu_stricto_1* in groups with graded CPC in diets (**Figure 8**).

**Figure 9:**
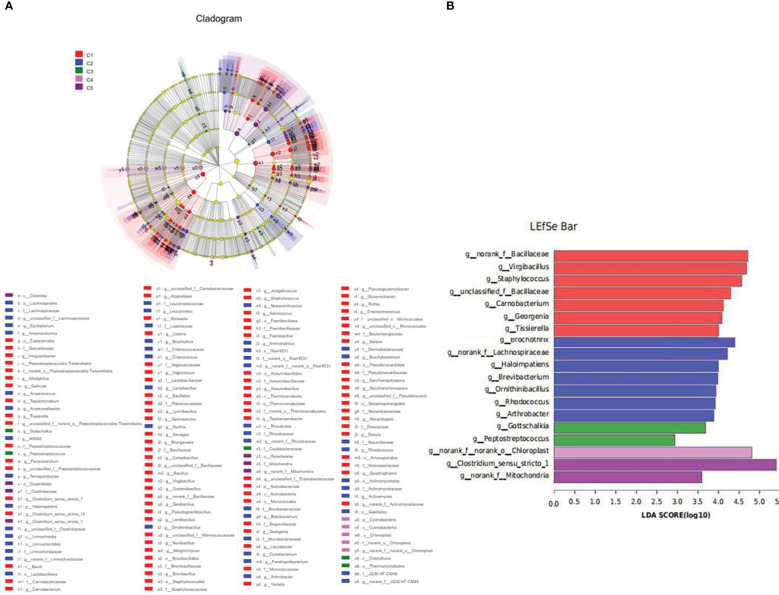
Inter-group variation in the relative abundance of intestinal microbial communities at the genus level. **(A)** Cladogram from Lefse. **(B)** LDA score from Lefse-PICRUSt.

LEfSe analysis revealed that the dominant bacterial genera in FM were *norank_f_Bacillaceae, virgibacillus, Staphylococcus, unclassified_f_Bacillaceae*, *Corynebacterium*, *Georgenia*, and *Tissierella*, and the main bacterial genera in CPC100% were *Clostridium_sensu_stricto_1* (**Figure 9**).

## 4 Discussion

### 4.1 Growth performance

Previous studies have demonstrated that dietary fishmeal can be partially replaced by CPC in carnivorous fish species without negative effects on growth performance (11, 25–27). It has been reported that CPC can replace up to 50% of dietary fishmeal (40% fishmeal in the control group) for largemouth bass (*Micropterus salmoides*) with no detrimental effects on growth performance, but a substitution rate of 75% for dietary fishmeal (fishmeal level: 10%) resulted in reductions in weight gain, feed efficiency, and condition factor (13). No adverse effects on growth were observed in juvenile golden pompano (*Trachinotus ovatus*) when 60% dietary fishmeal was replaced by CPC with 13.6% fishmeal and 20.4% CPC in the diet (28). For rainbow trout, a recent study showed that replacing 50% dietary fishmeal (40% fishmeal in the control group) by concentrated dephenolized cottonseed protein (CDCP) had no significant adverse effects on growth performance, survival rate, or feed utilization (17). However, there was no higher substitution level of fishmeal by CDCP in that study (17). In the present study, no significant detrimental effects on growth performance or feed intake were observed in rainbow trout when 75% dietary fishmeal was replaced by 22.5% CPC and only 7.5% fishmeal in the diet, indicating that CPC has the potential to replace fishmeal at a higher inclusion level. However, the significantly reduced feed intake and weight gain in the fishmeal-free diet based on CPC (CPC100%) suggests that it is still challenging to completely replace dietary fishmeal by CPC.

### 4.2 Metabolism

In the present study, no significant difference was observed in the proximate composition of whole-body fish when dietary fishmeal was replaced by graded CPC. However, the significant changes in contents of blood metabolites indicated alterations in metabolism in rainbow trout fed graded CPC. In this study, an increased substitute rate of fishmeal by CPC resulted in elevation of serum alanine aminotransferase (ALT) activity. ALT is a widely used indicator for hepatic function and metabolic syndrome (29–31). The increased serum ALT in rainbow trout fed graded CPC suggests that the inclusion of CPC in the diet could result in potential adverse effects on liver health. Previous studies also showed that detrimental effects of plant protein sources for fishmeal replacement could be due to the lack of cholesterol in plant proteins (9, 32). It has been reported that high inclusion levels of plant proteins in diets could reduce the level of blood cholesterol and result in hypocholesterolemia in carnivorous fish species (33, 34). In the present study, complete replacement of fishmeal by CPC resulted in reduced total cholesterol in serum. Further targeted metabolomic analysis for free sterols in serum of rainbow trout revealed no significant difference in free cholesterol of serum between the control group and fishmeal-free diet group based on CPC. However, the decreases of ergosterol, lathosterol, and cholestanol and increases of desmosterol and 7-dehydrocholesterol in serum of the fishmeal-free diet group based on CPC demonstrated an alteration in sterol metabolism, indicating that the free cholesterol level in serum could be balanced through metabolic regulation. Some studies have shown that supplementation of cholesterol in high plant protein diets could improve feed intake and growth performance in carnivorous fish species, but the effects of cholesterol supplementation varied with plant proteins (32, 34–36). Therefore, it is essential to investigate the effects of cholesterol supplementation in CPC-based diets on growth and feed intake in rainbow trout in the future.

In this study, complete replacement of fishmeal by CPC resulted in reductions of some free amino acids (e.g., lysine, methionine) in the serum of rainbow trout, although amino acids in diets were balanced by supplementation of crystalline amino acids. The reason for this could be the decreases in feed intake and digestibility. Previous studies in other carnivorous fish species also observed reduced feed intake and digestibility in fish fed high CPC. For example, dietary fishmeal replacement by CPC caused decreased feed intake and apparent digestibility in pearl gentian groupers (*Epinephelus fuscoguttatus*♀ × *E. lanceolatus*♂) (25). Further study is needed for efficient use of CPC in aquafeed through improving feed intake and digestibility.

### 4.3 Intestinal health

High inclusion of CPC in diets can result in impaired intestinal health of carnivorous fish species. Previous research on largemouth bass (*Micropterus salmoides*) revealed that 75% dietary fishmeal replacement induced impaired intestinal morphology, including reduced villus height and width (13). In rainbow trout, it has been reported that 50% fishmeal protein substitution by CPC showed no detrimental effects on intestinal morphology (17). However, the effects of dietary fishmeal replacement by CPC at higher inclusion levels on intestinal morphology were not reported in that study. In the present study, decreased villus height and width of the distal intestine was observed in rainbow trout fed diets with 75% fishmeal substitution, indicating that high inclusion levels of CPC could result in impaired intestinal morphology.

It is widely accepted that cytokines play important roles in the regulation of the immune system in vertebrates, and pro- and anti-inflammatory cytokines have been used as markers in assessment of intestinal health of fish (11, 37). In this study, 75% dietary fishmeal replacement induced upregulation of pro-inflammatory cytokines including IL1β, IL8, and TNFα, indicating an elevation of the inflammatory response. The results were in line with previous studies on hybrid grouper in which promotion of intestinal inflammation was found in the fish fed diets containing graded CPC (14, 25). The reason could involve the possible persistence of certain anti-nutritional factors.

In the present study, dietary fishmeal replacement by CPC reduced bacterial diversity in the intestine and altered the composition of the gut microbiota. Similarly, previous studies on carnivorous fish species showed reduction of bacterial diversity by CPC substitution (11, 12, 28). Meanwhile, fishmeal replacement by CPC decreased Actinobacteriota abundance and increased Firmicutes abundance in the present study. The results were in agreement with previous research where increased Firmicutes was observed in largemouth bass fed diets containing graded CPC (11). However, the decreased Actinobacteriota abundance was not found in other studies on CPC substitution, where alterations in Proteobacteria were often observed in largemouth bass (38). The discrepancy could be due to variation in fish species and feed formulations. At the genus level, reduced *Bacillaceae* was observed with graded CPC substitution in the present study. Previous studies demonstrated that *Bacillus* supplementation exhibited a variety of beneficial properties for carnivorous fish fed diets containing high levels of plant protein (e.g., soybean meal), including growth promotion, improvement in immune response, and general health (39–42). Therefore, the reduced Baci, llaceae could be responsible for the impaired intestinal health in experimental fish, and *Bacillus* may improve utilization of CPC-based diets for rainbow trout and other carnivorous fish species.

In conclusion, this study systematically evaluated CPC as a fishmeal alternative in the diets of rainbow trout, and the suggested substitution rate of fishmeal by CPC should be less than 75%. Excessive substitution of fishmeal by CPC and less than 75 g fishmeal/kg in the diet caused suppression of growth, alterations in blood metabolites, and impairment of intestinal health.

## Data availability statement

The raw sequences based on 16S rRNA gene can be found in the NCBI Sequence Read Archive (SRA) (Accession Number: PRJNA841419).

## Ethics statement

The animal study was reviewed and approved by Committee for the Welfare and Ethics of Laboratory Animals of Heilongjiang River Fisheries Research Institute of Chinese Academy of Fishery Sciences (CAFS).

## Author contributions

YL designed the study and wrote the manuscript. SM, HC, and GQ conducted the feeding trial and analyzed experimental data. WL, SL, DW, CW, and SH provided technical assistance. HL reviewed the manuscript. All authors contributed to the article and approved the submitted version.
