# Supplementary material for: Effects of replacing fishmeal with cottonseed protein concentrate on growth performance, blood metabolites, and the intestinal health of juvenile rainbow trout (Oncorhynchus mykiss)
Source: Front Immunol. 2022 Dec 21;13:1079677. doi: 10.3389/fimmu.2022.1079677 (PMC9811179; doi:10.3389/fimmu.2022.1079677)
Supplement: Supplementary file 1 [file DataSheet_1.docx]

Table S1 Slope values for reference and target genes

| Gene | Slope value |
| --- | --- |
| β-actin | 0.0647 |
| C3 | 0.0777 |
| C4 | 0.0768 |
| IL1β | 0.0459 |
| IL8 | 0.0614 |
| TNF-α | 0.0626 |
| IL10 | 0.0477 |
| TGF-β | 0.0892 |
| OCLN | 0.0577 |
| TRIC | 0.0353 |
| ZO1 | 0.0798 |
| CLD1 | 0.0708 |

Table S2 Alpha diversity indices of the intestinal microbial community of juvenile rainbow trout fed diets containing graded cottonseed protein concentrate （Mean±S.E.）

| Item | C1 | C2 | C3 | C4 | C5 |
| --- | --- | --- | --- | --- | --- |
| sobs | 211.00±10.74^d^ | 196.80±12.60^cd^ | 160.40±7.99^bc^ | 142.40±12.28^b^ | 101.00±21.59^a^ |
| shannon | 3.23±0.03^b^ | 3.11±0.07^b^ | 1.99±0.31^a^ | 1.89±0.20^a^ | 1.36±0.38^a^ |
| ace | 230.27±13.34^c^ | 214.08±18.51^bc^ | 178.39±12.05^b^ | 166.16±16.40^b^ | 117.17±20.37^a^ |
| chao | 232.45±16.09^c^ | 212.48±17.41^bc^ | 175.76±10.93^b^ | 166.22±16.67^ab^ | 117.53±20.92^a^ |
| coverage | 99.90±0.02 | 99.91±0.02 | 99.93±0.02 | 99.91±0.01 | 99.94±0.01 |

Note: values with different superiscripts in the same row are significantly different (*P*＜0.05)


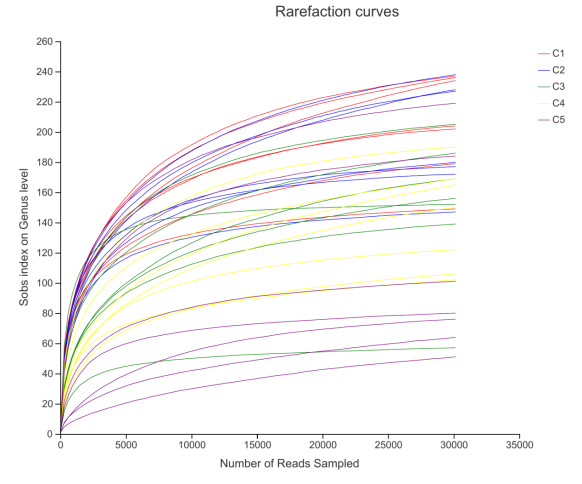

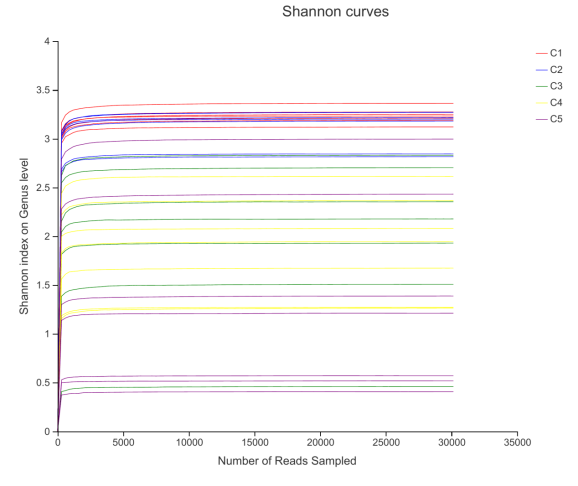


Figure S1 Rarefaction curve analysis of intestinal microbial diversity using the Sobs index (a) and the Shannon index (b) to assess operational taxonomic units (OTUs)

###
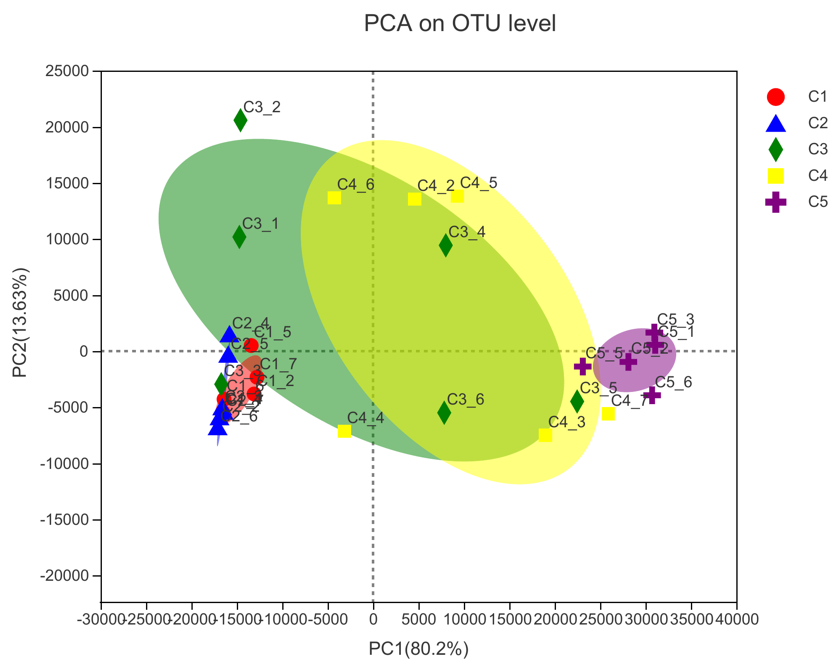


Figure S2 Principal Component Analysis（PCA）of the intestinal microbial communities


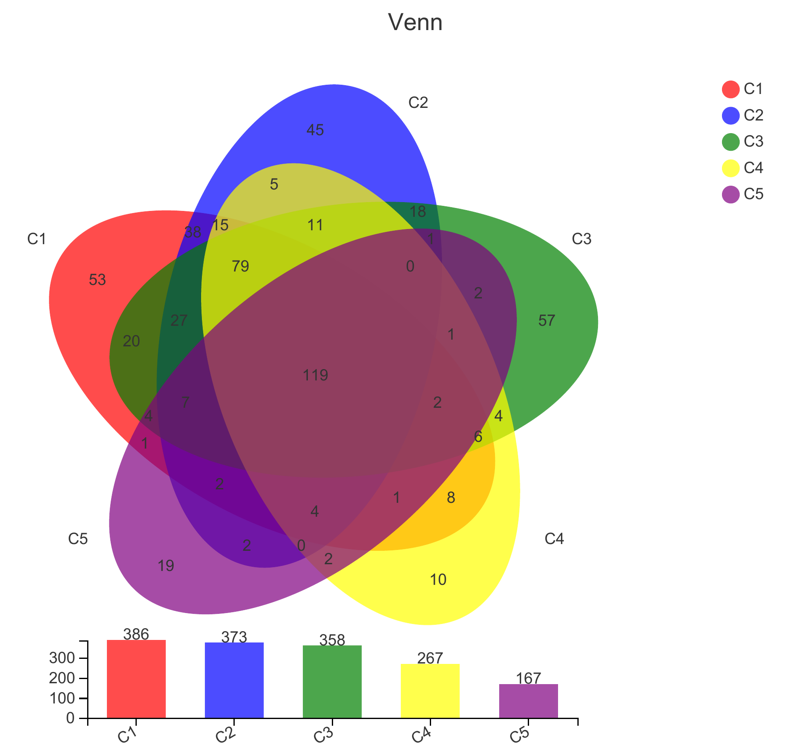


Figure S3 Venn diagram analysis of shared and unique OTUs between groups at the genus level
